# Association between pre-conceptional carbohydrate quality index and the incidence of gestational diabetes: the SUN cohort study

**DOI:** 10.1017/S000711452200157X

**Published:** 2023-02-28

**Authors:** Elena Fernández-González, Miguel Á. Martínez-González, Maira Bes-Rastrollo, David Suescun-Elizalde, Francisco Javier Basterra-Gortari, Susana Santiago, Alfredo Gea

**Affiliations:** 1 University of Navarra, Department of Preventive Medicine and Public Health, Pamplona, 31008, Spain; 2 Department of Endocrinology and Clinical Nutrition, Hospital Universitario Rey Juan Carlos, Móstoles, Madrid; 3 IdisNA, Pamplona, Spain; 4 Biomedical Research Network Center for Pathophysiology of Obesity and Nutrition (CIBEROBN), Carlos III Health Institute, Madrid, Spain; 5 Harvard TH Chan School of Public Health, Department of Nutrition, Boston, USA; 6 Complejo Hospitalario de Navarra, Department of Endocrinology and Nutrition, Pamplona, Spain; 7 University of Navarra, Department of Food Sciences and Nutrition, Pamplona, Spain

**Keywords:** Carbohydrates quality, Gestational diabetes, Cohort study, Pre-gestational diet, Primary prevention

## Abstract

The aim of the study was to investigate the association between pre-gestational carbohydrate quality index (CQI) and the incidence of gestational diabetes mellitus (GDM). Data from the ‘Seguimiento Universidad de Navarra’ (SUN) cohort were used, which includes 3827 women who notified at least one pregnancy between December 1999 and December 2019. We used a validated semi-quantitative 136-item FFQ to evaluate dietary exposures at baseline and at 10-year follow-up. The CQI was defined by four criteria: glycaemic index, whole-grain/total-grain carbohydrate, dietary fibre intake and solid/total carbohydrate ratio. We fitted generalised estimating equations with repeated measurements of the CQI to assess its relationship with incident GDM. A total of 6869 pregnancies and 202 new cases of incident GDM were identified. The inverse association between the global quality of carbohydrate and the development of GDM was not statistically significant: OR the highest *v*. the lowest CQI category: 0·67, 95 % CI (0·40, 1·10), *P*_for trend_ = 0·10. Participants at the highest CQI category and with daily carbohydrate amounts ≥50 % of total energy intake had the lowest incidence of GDM (OR = 0·29 (95 % CI (0·09, 0·89)) compared with those with the lowest quality (lowest CQI) and quantity (≤40 %). Further studies are needed to overcome the limitations of our study. Those studies should jointly consider the quality and the quantity of dietary carbohydrates, as the quality might be of importance, especially in women with a higher intake of carbohydrates.

Gestational diabetes mellitus (GDM) is defined as the alteration of the metabolism of hydrocarbons which meets established diagnostic criteria, it is first recognised during pregnancy and that it does not comply with the established diagnostic criteria for diabetes mellitus prior to gestation^(1)^. The prevalence of GDM has increased in recent years, especially due to the advanced age of pregnant women, the sedentary lifestyle and the increasing incidence of overweight and obesity^(2,3)^.

GDM is associated with several complications relevant to both the child (i.e. macrosomia, large for gestational age, prematurity, traumatic births and caesarean, metabolic alterations such as hypoglycaemia and hyperbilirubinemia, polyhydramnios, possibility of developing long-term alteration of the metabolism of hydrocarbons and metabolic syndrome) and the mother (i.e. pre-eclampsia and eclampsia, type 2 diabetes (DM2) and CVD)^(4–15)^.

Well-established risk factors for developing GDM are advanced maternal age, overweight and obesity and excessive weight gain in the index pregnancy, physical inactivity, ethnicity, history of macrosomic babies (birth weight 4000 g or more) or previous unexplained perinatal loss^(16,17)^ or birth of a malformed infant^(18)^, previous diagnosis of GDM or other alterations of glucose metabolism, multiple gestation, polycystic ovarian syndrome^(19)^, or family history of DM2^(20–22)^. Moreover, genetic risk has also been described^(23)^.

Diet has also been postulated as a modifiable risk factor for GDM^(24)^. Previous articles have reported preventive association between healthful dietary patterns, in particular the Mediterranean diet, and GDM risk^(25–29)^. Other several nutritional factors have been found to increase the risk of developing GDM: high intake of animal fat^(30)^, high cholesterol and eggs consumption (≥7 eggs/week)^(31)^, high intake of heme iron^(32)^ and frequent consumption of fried food, mainly out of home^(33)^. Previous analysis within our cohort found a higher incidence of GDM associated with higher levels of red and processed meat consumption, Fe intake and high levels of fast-food consumption^(34,35)^.

Important dietary factors potentially related to the risk of GDM are both carbohydrate quantity and quality^(36)^. Four previous studies found that a lower fibre intake, a higher glycaemic load, glycaemic index, or a diet low in carbohydrates but high in proteins and fats from animal products may increase the risk of developing gestational diabetes^(37–40)^. However, none of these studies assessed a multidimensional index of carbohydrate quality. In this context, we aimed to analyse the overall quality of carbohydrates ingested before pregnancy, by means of a composite multidimensional index, previously used in other studies, considering simultaneously four characteristics: Glycaemic Index (GI), fibre content, solid or liquid form, and the degree of processing, namely the previously described carbohydrate quality index (CQI)^(41–45)^. We related this in association with GDM risk in a prospective cohort: the Seguimiento Universidad de Navarra (SUN) Project.

## Subjects and methods

### Study population

The SUN Project is a Spanish, prospective, multipurpose, dynamic cohort study composed of university graduates. The main objective was to investigate the association between diet, health conditions and multiple chronic diseases. The recruitment has been permanently opened since 1999. The design, follow-up methods and validated questionnaires have been formerly published^(46,47)^. A mailed questionnaire regarding dietary habits, lifestyles and health conditions is used to invite graduates to participate in the study, and a first voluntary response is assumed as informed consent to participate. Every 2 years, the information about diet, lifestyle, risk factors and medical conditions is updated with further follow-up questionnaires. The study protocol was performed as directed by the Declaration of Helsinki and approved by the Institutional Review Board of the University of Navarra. The SUN Project is registered at clinicaltrials.gov as NCT02669602. All questionnaires are available at https://www.unav.edu/en/web/departamento-de-medicina-preventiva-y-salud-publica/proyecto-sun/informacion-para-investigadores.

**Fig. 1.**
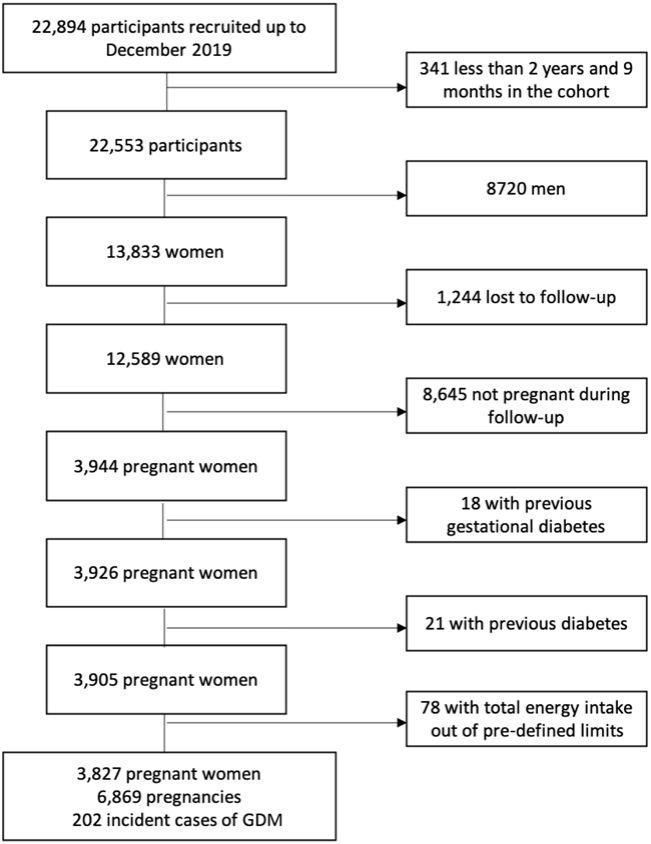
Flow chart of participants. GDM, gestational diabetes mellitus

For the present analysis, we used the available database as of December 2019, accounting for 22 894 participants. The flow chart of participants is presented in Fig. 1. We excluded 341 subjects as they had been less than 2 years and 9 months in the cohort, to make sure that all the participants had been in the cohort time enough to complete at least the first follow-up questionnaire, and 8720 men. Up to the remaining 13 833 women, 12 589 women completed at least one follow-up questionnaire and 1244 were lost to follow-up. Therefore, the overall retention in the cohort was 91 %. Subsequently, other 8645 were excluded as they reported no pregnancies during follow-up. Women with a diagnosis of GDM previous to baseline were also excluded (*n* 18). Additionally, participants who reported a medical diagnosis of diabetes (both type 1 or 2) and/or they were being treated with insulin and/or oral antidiabetics at baseline were excluded (*n* 21). Women with implausible levels of total energy intake (lower than percentile 1 or higher than percentile 99) were also excluded in order to assure their correct fulfilment of the dietary information (*n* 78). Therefore, the final sample included 3827 pregnant women, with a total of 6869 incident pregnancies and 202 incident GDM cases.

### Assessment of exposure: carbohydrate quality index

A semi-quantitative FFQ with 136 food items was used to assess the dietary exposures at baseline and after 10 years of follow-up^(48,49)^. It has been repeatedly validated in Spain, and it has shown good validity for assessing carbohydrate intake^(50)^. There were nine options for the average frequency of consumption (never, 1 to 3 times/month, once/week, 2–4 times/week, 5–6/week, once daily, 2–3 times daily, 4–6 times daily, and 6 or more times daily). For this purpose, nutrient composition contained in the portion size specified for each food (typical portion size) was multiplied by the frequency of consumption.

Baseline dietary intake data were used to compute the CQI as proposed by Zazpe *et al*.^(51)^. The CQI considers four criteria: (a) the GI (inversely weighted); (b) ratio of carbohydrates from whole grains to carbohydrates from total grains (whole grains, refined grains and its products); (c) dietary fibre intake (g/d) and (d) ratio of solid carbohydrates to total (solid and liquid). GI was derived for each food and beverage item in the FFQ using reference tables that used glucose as the reference food.

For each of these four components, participants were categorised into quintiles (score from 1 to 5, except for GI, that was weighted from 5 to 1), with higher values meaning better quality of each element. We added up the total score that ranged from 4 to 20. Finally, we categorised participants in three categories of the CQI: 4–9, 10–14 and 15–20 (see Supplementary Table 1).

### Ascertainment of the outcome: gestational diabetes

Pregnant women who reported GDM diagnosis for the first time were asked to submit further information: medical reports, a previous diagnosis of diabetes, written confirmation of the date of the diagnosis, highest fasting glucose, glycated haemoglobin during pregnancy and oral glucose tolerance test as well as insulin use during the pregnancy. This information was verified by a medical team that was blinded to any exposure of the participants, and only the cases that this team confirmed were considered in the analysis.

There is no universal criterion for the diagnosis of GDM. Several protocols with different cut-off points are used for diagnosis after overload, which is also performed with different amounts of ingested glucose. An endocrinologist blinded to the information about dietary habits examined the information provided by women (additional questionnaires and medical reports) to confirm the diagnosis of incident gestational diabetes. The most common diagnosis criteria for GDM in Spain were those with 100 g oral glucose tolerance test with the cut-offs of Carpenter and Coustan or the cut-offs from the National Diabetes Data Group after a positive 50 g glucose challenge test.

### Assessment of covariates

In the baseline questionnaire, other covariates were considered: social demographic variables (age), anthropometric measures (weight and BMI), health-related habits (physical activity, hours sitting down/d, smoking habits, total energy intake, adherence to Mediterranean diet, adherence to special diets, fast-food and snack consumption) and medical history (medication intake, parity and family history of DM2, CVD or hypertension).

A specific study with a subsample of this cohort pointed out a high validity of the self-reported weight and BMI^(52)^. Physical activity was evaluated according to a validated questionnaire and was measured in metabolic equivalent tasks (MET-h/week)^(53)^. It was obtained by multiplying the duration of each activity in h/week by its characteristic energy expenditure. Adherence to the Mediterranean diet was computed using the nine-item score proposed by Trichopoulou *et al*.^(54)^ but excluding cereals item to avoid overlapping with the main exposure, and alcohol intake, that was considered separately.

### Statistical analysis

To describe baseline characteristics of the participants according to categories of CQI, we used percentages for categorical variables and means and standard deviations for continuous variables. To assess the relationship between CQI and the incidence of GDM, generalised estimating equations were fitted, assuming a binomial distribution, a logit link function, an exchangeable correlation matrix, and with a robust variance estimator, obtaining OR with 95 % CI. Each pregnancy was individually considered. For a woman who developed GDM during follow-up, no further pregnancies were considered thereafter. For those pregnancies occurring after more than 10 years of follow-up, exposure in the 10-year questionnaire was considered. Four models were fitted with increasing levels of adjustment: (a) unadjusted model; (b) adjusted for age at the first pregnancy at the cohort (continuous), BMI (continuous); (c) additionally adjusted for parity (0, 1, 2, 3 or more), family history of DM (yes/no), leisure-time physical activity (MET-h/week) (tertiles), television viewing (h/d) (continuous), smoking habit (never smokers, former smokers and current smokers), total energy intake (continuous), adherence to Mediterranean diet (three categories), fast-food consumption (three categories), snacking between meals (yes/no), following special diet at the baseline (yes/no); and (d) additionally adjusted for alcohol intake (abstainer, less than 10 g/d, 10–20 g/d and more than 20 g/d), CVD prior to enrolment (including CHD and stroke: yes or no) and hypertension prevalence (yes or no). Test for linear trend was performed using the median value of CQI for each category of CQI as a continuous variable in the model. The association between tertiles of individual components of the CQI and the incidence of GDM was also assessed. Several sensitivity analyses were performed to test the robustness of the results. Additionally, a joint analysis was conducted to jointly consider the quality (three categories of CQI) and the quantity of dietary carbohydrates (≤40 %, >40–<50 % and ≥50 %). Tests were two-sided, and *P* < 0·05 was considered statistically significant. The analyses were performed with STATA software version 16.1.

## Results

Among 6869 incident pregnancies, 202 incident cases of new-onset GDM were identified (2·94 %). Baseline characteristics of subjects according to categories of baseline CQI are shown in Table 1. Participants in the lowest CQI category were younger and had their first pregnancy at a younger age, had a lower level of physical activity, and were more likely to be current smokers. Moreover, their total energy intake was lower, and they consumed lower quantities of total fat intake, MUFA, PUFA, SFA, and *trans*-fatty acid intake, and their adherence to the Mediterranean dietary pattern was lower.

**Table 1.** Baseline characteristics of participants according to baseline categories of carbohydrate quality index (Mean values and standard deviations or percentages)

	Carbohydrate quality index					
	Low		Moderate		High	
	Mean	SD	Mean	SD	Mean	SD
CQI (range)	4–9		10–14		15–20	
*n*	1150		1954		723	
Age at baseline (years)	27·7	4·4	28·2	4·6	28·6	4·7
Age at first incident pregnancy (years)	34·4	4·1	34·6	4·3	34·8	4·5
Incidence of gestational diabetes						
%	5·8		5·2		4·7	
BMI (kg/m^2^)	21·3	2·7	21·4	2·6	21·5	2·6
Parity status at baseline						
Nulliparous						
%	84·6		83·3		84·5	
One previous pregnancy						
%	8·5		8·9		9·5	
Two previous pregnancies						
%	4·1		4·4		4·2	
Three or more previous pregnancies						
%	2·8		3·4		1·8	
Family history of DM2						
%	8·0		10·6		10·5	
Physical activity (MET-h/week)	15·1	16·7	19·7	21·2	23·5	21·8
Television viewing (h/d)	1·8	1·3	1·7	1·2	1·6	1·2
Smoking status						
Never smokers						
%	52·4		57·6		60·2	
Former smokers						
%	18·1		18·4		19·8	
Current smokers						
%	29·5		24·1		20·1	
Alcohol intake (pre-conceptional) (g/d)	4·1	5·8	3·9	5·2	3·9	4·8
Total energy intake (kJ/d)	9640	2761	10661	3213	11326	3339
Carbohydrate intake (% energy)	42·3	7·0	43·3	7·2	44·6	7·1
Glycaemic index	52·2	4·2	51·83	4·26	50·7	4·1
Fibre intake (g/d)	19·5	6·1	29·90	10·67	42·7	14·3
Solid carbohydrate/total	0·83	0·09	0·87	0·07	0·90	0·05
Carbohydrates from whole grains/total	0·00	0·03	0·07	0·13	0·22	0·18
Protein intake (% energy)	17·8	3·1	18·0	3·1	18·6	3·2
Fat intake (% energy)	38·7	6·3	37·5	6·5	35·8	6·4
MUFA intake (% energy)	16·4	3·6	16·0	3·7	15·2	3·5
PUFA intake (% energy)	5·5	1·7	5·3	1·6	5·0	1·5
SFA intake (% energy)	13·6	3·1	12·8	3·2	11·7	3·0
TFA intake (% energy)	0·42	0·19	0·37	0·18	0·33	0·16
Mediterranean dietary pattern (0–7)*	3·2	1·5	4·2	1·6	5·2	1·6
Fast-food consumption (g/d)	25·5	18·9	25	22·3	23·6	18·7
Special diet						
%	3·7		6·5		12·2	
Snacking						
%	46·4		41·8		36·1	
Prevalence of hypertension						
%	1·8		1·4		3·9	
Prevalence of CVD						
%	0·09		0·41		0·14	

* Adherence to the Mediterranean dietary pattern was computed using the nine-item score proposed by Trichopoulou *et al.*^(51)^ excluding cereals and alcohol intake.

In the fully adjusted model (Table 2), for the comparison between the highest CQI category *v*. the lowest, the OR was 0·67, suggesting an inverse association, but the 95 % CI was too wide to reach a definitive conclusion (95 % CI (0·40, 1·10)). Increasing adherence to the CQI was inversely associated with the incidence of GDM, but the results did not reach the conventional level for statistical significance: the *P*_for trend_ was 0·102 and the OR (95 % CI) for a two-point increment in the CQI was 0·96 (95 % CI (0·86, 1·07)) in the fully adjusted model.

**Table 2.** Association between carbohydrate quality index and the incidence of gestational diabetes (Odd ratio and 95 % confidence intervals)

	Carbohydrate quality index								
			Moderate (10 to 14)		High (15 to 20)		*P*_for trend_	Two-point increment	
	Low (4 to 9)		OR	95 % CI	OR	95 % CI		OR	95 % CI
Cases/pregnancies	65/2091		104/3527		33/1251				
Crude model	1	Ref.	0·94	0·69, 1·30	0·84	0·55, 1·29	0·051	1·00	0·92, 1·10
Multivariable model 1	1	Ref.	0·92	0·67, 1·27	0·82	0·53, 1·26	0·044	1·00	0·91, 1·09
Multivariable model 2	1	Ref.	0·80	0·58, 1·12	0·66	0·40, 1·09	0·105	0·96	0·86, 1·07
Multivariable model 3	1	Ref.	0·80	0·57, 1·11	0·67	0·40, 1·10	0·102	0·96	0·86, 1·07
Multivariable model 4	1	Ref.	0·86	0·61, 1·19	0·75	0·47, 1·20	0·059	0·98	0·89, 1·08

Model 1: Adjusted for age at the first pregnancy at the cohort (continuous), BMI (continuous).

Model 2: Additionally adjusted for baseline parity (0, 1, 2, 3 or more), family history of DM (yes/no), leisure-time physical activity (metabolic equivalent-h/week, tertiles), television viewing (h/d, continuous), smoking habit (never smokers, former smokers and current smokers), total energy intake (continuous), adherence to Mediterranean diet (three categories), fast-food consumption (three categories), snacking between meals (yes/no) and following special diet at baseline (yes/no).

Model 3: Additionally adjusted for alcohol intake (0, >0 to 10 g/d, >10 to 20 g/d and >20 g/d), CVD (yes or no) and hypertension prevalence (yes or no).

Model 4: Excluding Mediterranean diet from model 3.

Table 3 shows the association between the incidence of GDM and each of the four components of the CQI. We found that none of the CQI components separately was significantly associated with a lower incidence of developing GDM in the fully adjusted model, additionally adjusted for all the other components. The lower OR was found for the lowest tertile of GI (0·80, 95 % CI (0·53, 1·20)), and the highest tertile of carbohydrates from whole grain to carbohydrates from total grain (0·81, 95 % CI (0·57, 1·13)).

**Table 3.** OR and 95 % CI of incident gestational diabetes mellitus by tertiles of glycaemic index, fibre intake, ratio of solid carbohydrates/total carbohydrates and ratio of carbohydrates from whole grains to carbohydrates from total grains (Odd ratio and 95 % confidence intervals)

Glycaemic index			T2		T1		*P*_for trend_
	T3		OR	95 % CI	OR	95 % CI	
Median							
Cases/pregnancies	77/2271		66/2271		59/2327		
Multivariable model 3	1		0·84	0·59, 1·18	0·74	0·51, 1·06	0·004
Multivariable model 4	1	Ref	0·87	0·61, 1·25	0·80	0·53, 1·20	0·727
Fibre intake	T1		T2		T3		
Median							
Cases/pregnancies	61/2350		72/2273		69/2246		
Multivariable model 3	1	Ref	1·10	0·76, 1·60	0·95	0·58, 1·56	0·012
Multivariable model 4	1	Ref	1·15	0·79, 1·68	1·01	0·60, 1·70	0·518
Solid carbohydrates/total	T1		T2		T3		
Median							
Cases/pregnancies	74/2280		65/2338		63/2251		
Multivariable model 3	1	Ref	0·84	0·59, 1·20	0·82	0·56, 1·18	0·004
Multivariable model 4	1	Ref	0·88	0·61, 1·27	0·89	0·60, 1·32	0·744
Carbohydrates from whole grains/total	T1		T2		T3		
Median							
Cases/pregnancies	128/3950		13/676		61/2243		
Multivariable model 3	1	Ref	0·54	0·29, 0·98	0·80	0·57, 1·10	0·445
Multivariable model 4	1	Ref	0·55	0·30, 1·02	0·81	0·57, 1·13	0·176

CQI, carbohydrate quality index.

Model 3: Adjusted for age at the first pregnancy at the cohort (continuous), BMI (continuous), baseline parity (0, 1, 2, 3 or more), family history of DM (yes/no), leisure-time physical activity (metabolic equivalent-h/week, tertiles), television viewing (h/d, continuous), smoking habit (never smokers, former smokers and current smokers), total energy intake (continuous), adherence to Mediterranean diet (three categories), fast-food consumption (three categories), snacking between meals (yes/no), following special diet at baseline (yes/no), alcohol intake (0, >0 to 10 g/d, >10–20 g/d and >20 g/d), CVD (yes or no) and hypertension prevalence (yes or no).

Model 4: Additionally adjusted for the other three components of the CQI.

Table 4 shows the results of the joint analysis of the quality and quantity of carbohydrates and incidence of GDM. Whereas there was no statistically significant interaction between both factors (*P* = 0·62), those participants with the highest CQI and with daily carbohydrate amounts > 50 % of total energy intake exhibited a significantly lower incidence of GDM: OR 0·29, 95 % CI (0·09, 0·89) as compared with those with the lowest quality and quantity (<40 %).

**Table 4.** Association between carbohydrate quality and quantity and the incidence of gestational diabetes (Odd ratio and 95 % confidence intervals)

			Carbohydrate quality index					
			Low		Moderate		High	
			OR	95 % CI	OR	95 % CI	OR	95 % CI
Carbohydrate intake (% E)	≤ 40 %	Cases/pregnancies	26/726		34/1121		12/337	
			1	Ref.	0·65	0·38, 1·12	0·70	0·33, 1·50
	40–50 %	Cases/pregnancies	32/1131		51/1826		17/633	
			0·70	0·41, 1·20	0·60	0·36, 1·00	0·53	0·26, 1·07
	≥ 50 %	Cases/pregnancies	7/234		19/580		4/281	
			0·71	0·29, 1·69	0·67	0·35, 1·30	0·29	0·09, 0·89

Adjusted for age at the first pregnancy at the cohort (continuous), BMI (continuous), baseline parity (0, 1, 2, 3 or more), family history of DM (yes/no), leisure-time physical activity (metabolic equivalent-h/week, tertiles), television viewing (h/d, continuous), smoking habit (never smokers, former smokers and current smokers), total energy intake (continuous), adherence to Mediterranean diet (three categories), fast-food consumption (three categories), snacking between meals (yes/no), following special diet at the baseline (yes/no), alcohol intake (0, >0 to 10 g/d, >10–20 g/d and >20 g/d), CVD (yes or no) and hypertension prevalence (yes or no).

*P*_for interaction_ = 0·62.

Table 5 shows the results of the sensitivity analyses. Compared with the main analysis, results of the OR were not substantially different in any of the analysed scenarios, although in some cases the 95 % CI was narrower and more suggestive of an inverse association.

**Table 5.** Sensitivity analyses (Odd ratio and 95 % confidence intervals)

	Cases/pregnancies	C1		C2		C3		*P*_for trend_
				OR	95 % CI	OR	95 % CI	
Main analysis	202/6869	1	Ref.	0·80	0·57, 1·11	0·67	0·40, 1·10	0·102
a) Including only women who did not have pregnancy before entering the cohort	173/5993	1	Ref.	0·85	0·59, 1·21	0·63	0·36, 1·11	0·056
b) Excluding obese women at baseline	195/6786	1	Ref.	0·84	0·60, 1·18	0·71	0·43, 1·19	0·083
c) Excluding pregnancies of women over 40 years of age	192/6506	1	Ref.	0·81	0·57, 1·14	0·68	0·41, 1·14	0·145
d) Considering only the first pregnancy during follow-up	187/3827	1	Ref.	0·79	0·56, 1·13	0·65	0·30, 1·06	0·076
e) Considering baseline exposure	202/6869	1	Ref.	0·78	0·56, 1·09	0·68	0·41, 1·13	0·112
f) Both d) and e)	187/3827	1	Ref.	0·79	0·56, 1·13	0·65	0·40, 1·06	0·076
g) Adjusting for baseline age instead of age at first considered pregnancy	202/6869	1	Ref.	0·80	0·57, 1·12	0·65	0·39, 1·08	0·353
h) Adjusting for age at each pregnancy instead of age at first considered pregnancy	202/6869	1	Ref.	0·80	0·57, 1·12	0·69	0·41, 1·14	0·302
i) Adjusting for baseline age and age at each pregnancy instead of age at first considered pregnancy	202/6869	1	Ref.	0·82	0·59, 1·14	0·66	0·40, 1·10	0·216
j) Additionally adjusting for fat intake	202/6869	1	Ref.	0·80	0·57, 1·12	0·68	0·41, 1·12	0·095

Adjusted for age at the first pregnancy at the cohort (continuous), BMI (continuous), baseline parity (0, 1, 2, 3 o more), family history of DM (yes/no), leisure-time physical activity (metabolic equivalent-h/week, tertiles), television viewing (h/d, continuous), smoking habit (never smokers, former smokers and current smokers), total energy intake (continuous), adherence to Mediterranean diet (three categories), fast-food consumption (three categories), snacking between meals (yes/no), following special diet at the baseline (yes/no), alcohol intake (0, >0 to 10 g/d, >10–20 g/d and >20 g/d), CVD (yes or no) and hypertension prevalence (yes or no).

## Discussion

In this prospective cohort study, pre-conceptional global quality of carbohydrate was not significantly associated with the incidence of GDM. We found an association between the incidence of GDM and a high-quality and high-quantity pre-conceptional dietary carbohydrates. Although most results were inconclusive, based on the CI and not only on the *P*-value^(55,56)^, results of this study suggest that further studies should jointly consider the quality and the quantity of dietary carbohydrates, as the quality might be of importance, in particular in women with a higher intake of carbohydrates.

Given the complications with which GDM is related for both the mother and the fetus, the ideal is to prevent its development by previously acting on modifiable risk factors such as diet.

In GDM, there is a situation of insulin resistance with dysfunctional β-cells, pathophysiological situation very similar to that which occurs in DM2. The relationship between the intake of high-quality carbohydrates with a lower incidence of DM2 has already been reported^(57)^. In fact, the main scientific societies propose some similar recommendations in the dietary management of both DM2 and GDM^(58,59)^ valuing the quality of ingested carbohydrates more than a fixed distribution of macronutrients that, by restricting the amount of carbohydrates in the diet, could lead to a compensatory increase in the percentage of ingested fat and thus insulin resistance^(60)^.

The Nurses’ Health Study II found that a low-carbohydrate dietary pattern with energy replacement from protein and fat from animal food sources may lead to an increased risk of GDM^(40,61)^. The Australian Longitudinal Study on Women’s Health (ALSWH) showed that habitual diet low in carbohydrates and high in fat and protein was associated with a higher incidence of GDM^(39)^.

Concerning the carbohydrate quality, dietary total fibre intake, and in particular fruit fibre, seems to reduce the risk of GDM^(37,39)^. A high glycaemic load diet might increase the risk of GDM^(37,39)^. The value of dietary glycaemic load in terms of postprandial glycaemic response has been described in both healthy subjects^(62)^ and overweight patients with DM2^(63)^. However, in women with GDM, the results have been inconclusive^(64,65)^.

Also a higher consumption of French fries might increase the risk of GDM, since it is mainly a food with a high glycaemic index and that after the frying process it will contain advanced glycosylation products that will favour the development of insulin resistance^(66)^. Moreover, regular consumption of sugar-sweetened cola (which provides with considerable rapidly absorbable sugars) had been associated with a higher incidence of GDM^(67,68)^.

Considering the whole dietary pattern, several cohorts have found a lower incidence of GDM with greater adherence to Mediterranean diet^(25–28,69–71)^. Carbohydrates in the Mediterranean diet are mainly from whole grain, avoiding the consumption of those with rapid absorption and high GI like the sugar-sweetened drinks; it is rich in fibre, with the high consumption of fruits and vegetables, legumes, and nuts. Other dietary patterns such as the DASH (Dietary Approaches to Stop Hypertension), and the AHEI (Alternate Healthy Eating Index) have also been associated with a lower incidence of GDM^(25)^.

Regarding intervention studies before or during pregnancy, the results are inconsistent^(72)^ or inconclusive^(73,74)^. Some interventions during pregnancy on diet and physical activity achieved lower incidences of GDM^(75–77)^. Moreover, interventions during pregnancy to increase adherence to Mediterranean diet also reduced the incidence of GDM^(78,79)^. Further randomised trials are needed.

Regarding biological plausibility, several potential mechanisms have been described in the literature. Dietary fibre can delay gastric emptying by reducing appetite and slowing and decreasing glucose absorption and insulin response^(80,81)^. It can lead to a lower total daily energy intake which in turn will reduce fat and improve insulin sensitivity. In addition, it can reduce inflammation, aid in lipid control, and have antioxidant effects.

Data for whole grains alone are limited due to disparities in the definition of whole food in previous epidemiological studies. The American Nutrition Society supports the consumption of foods rich in fibre from cereals and whole grains to reduce the risk of DM2, obesity and CVD^(82)^.

The liquid form of carbohydrates favours faster absorption, less satiety, greater total energy intake and higher postprandial blood glucose elevation, with respect to carbohydrates ingested in solid form. Increased sugar consumption can also lead to deterioration of pancreatic β-cells, possibly due to the accumulation of reactive oxygen species^(83)^.

Finally, the high glycaemic index of food implies a faster and greater increase in blood glucose and insulin levels after ingestion. This increase in insulin stimulates the absorption of nutrients by the cell and lipogenesis and inhibits the production of glucose in the liver^(84)^.

The correlation between fibre and whole-grain intake was not high in our study (0·263). Therefore, we assumed that we were not evaluating the same question twice, but that fibre intake will include that of other foods beyond those contained in whole grains.

Our study has some limitations. For the evaluation of the diet, a self-reported FFQ was used. In our study, participants with higher BMI (and consequently higher risk of GDM) might be more likely to follow an energy-restricted diet and also to under-report their intake of unhealthy food. However, the FFQ is the most reliable and valid tool to assess the diet in large epidemiological studies. It has been previously evaluated with good correlation between the data obtained from the FFQ and those from dietary records^(48–50)^. On the other hand, a particular variable to capture dieting was added as a covariate in the model, so potential differences or misreporting due to this factor were controlled for in the models.

As we have already indicated, the FFQ questionnaire was carried out at the beginning of the study and at 10 years of follow-up, but not during pregnancy. It is possible that by knowing their pregnancy status, participants might have modified their eating habits. However, the dietary pattern maintained prior to pregnancy seems to have a stronger influence on the development of GDM than the temporary changes in intake during pregnancy, which tend to affect specific foods more than the dietary pattern itself^(85–87)^.

We have used a pre-defined index that assesses the quality of carbohydrates in a wide manner^(41–45)^. However, it cannot encompass all aspects related to the intake of these macronutrients. In addition, there is the controversy regarding the definition of a whole-grain or carbohydrate-restricted diet, which makes comparison between studies difficult.

Another limitation is that we do not have the sufficient power to compare the effect of the CQI on each of the subgroups that would be created according to the severity of GDM.

In addition, women diagnosed with GDM with several of the different criteria accepted for it were included as incident cases. We also do not have data on weight gain during pregnancy, since the questionnaires were completed every 2 years.

In the study, we adjusted for multiple confounding factors; however, we cannot totally rule out the possibility of residual confounding.

We only included women with a first diagnosis of GDM, without accounting for recurrence in subsequent pregnancies. Those women could have changed their diet after the first GDM diagnosis and on the other hand, they will also be more likely to have GDM recurrence. To avoid that nulliparous women were overrepresented in the GDM group, we adjusted for parity in the multivariate analysis and also carried out sensitivity analyses restricting nulliparous women, obtaining results similar to the main analysis.

On the other hand, our cohort only included women with a high level of education. This limits the representativeness of the sample, but at the same time it allows to avoid several confounding factors with the use of restriction. Our results acquire relevance given the explanation by the possible pathophysiological mechanisms described and not so much by their representativeness or external validity. The characteristics of this population with a high educational level could also condition that the women included had in general a better quality of the diet due to knowledge of the negative effects of the ingestion of lower quality carbohydrates, and therefore there would be fewer differences between them, which could justify our results. It is possible that we did not find a significant reduction in risk due to lack of statistical power as the number of incident cases was low.

However, our study has a number of strengths: it is a cohort of prospective design with large numbers of participants followed for a long time, in which it has been possible to adjust for multiple confounding factors. These are participants with a high level of education, which allows greater precision of their self-reported data and better retention in the cohort (overall retention 91 %). Moreover, our medical team confirmed the diagnoses of the GDM cases reported.

Regarding age, a prominent risk factor for GDM^(88)^, we looked for the best adjustment option given the characteristics of our cohort. For this, we used the age of the first pregnancy in the cohort, therefore subsequent to the collection of data on dietary exposure. On the other hand, if we had used the age in the last pregnancy, we could create an inverse artificial relationship with the risk of gestational diabetes. In addition, we carried out several sensitivity analyses, adjusting for the age of entry into the cohort, the age at each pregnancy when doing a repeated-measures study, and the combined adjustment for the age at entry into the cohort and the age at each pregnancy. In each of the assumptions, the results of the analysis were very similar, so we assumed the option of adjusting for the age of the first pregnancy at follow-up, since it would be the one that would have the most relationship with baseline exposure.

In conclusion, in this Mediterranean prospective cohort, quality of dietary carbohydrates was not significantly associated with the incidence of GDM. Results suggest that a higher quality of dietary carbohydrates might be associated with lower incidence of GDM, in particular in women with a higher intake of carbohydrates. Future studies should jointly consider both the quality and the quantity of dietary carbohydrates. However, these studies must overcome the limitations of the current study, with a larger sample size and a dietary assessment closer to conception, to confirm the effect of the quality and quantity of dietary carbohydrates on the development of GDM.
